# Supplementary material for: Fluorescence shadow imaging of Hypsibius exemplaris reveals morphological differences between sucrose- and CaCl2-induced osmobiotes
Source: Sci Rep. 2024 May 23;14:11845. doi: 10.1038/s41598-024-61374-y (PMC11116526; doi:10.1038/s41598-024-61374-y)
Supplement: Supplementary file 3 — Supplementary Information 1. [file 41598_2024_61374_MOESM3_ESM.pdf]

Fluorescence Shadow Imaging of *Hypsibius exemplaris* Reveals Morphological Differences Between Sucrose- and $CaCl_2$-Induced Osmobiotes

Brendin B. Flinn[1], Hayden M. O'Dell[1], Kara M. Joseph[1], Amanda L. Smythers[2], David P. Neff[1], Leslie M. Hicks[2], Michael L. Norton[1], and Derrick R.J. Kolling[1,*]

1 Marshall University, Department of Chemistry, Huntington, WV, USA
2 University of North Carolina at Chapel Hill, Department of Chemistry, Chapel Hill, NC, USA
* Correspondence: kolling@marshall.edu; Tel.: (304) 696-2307

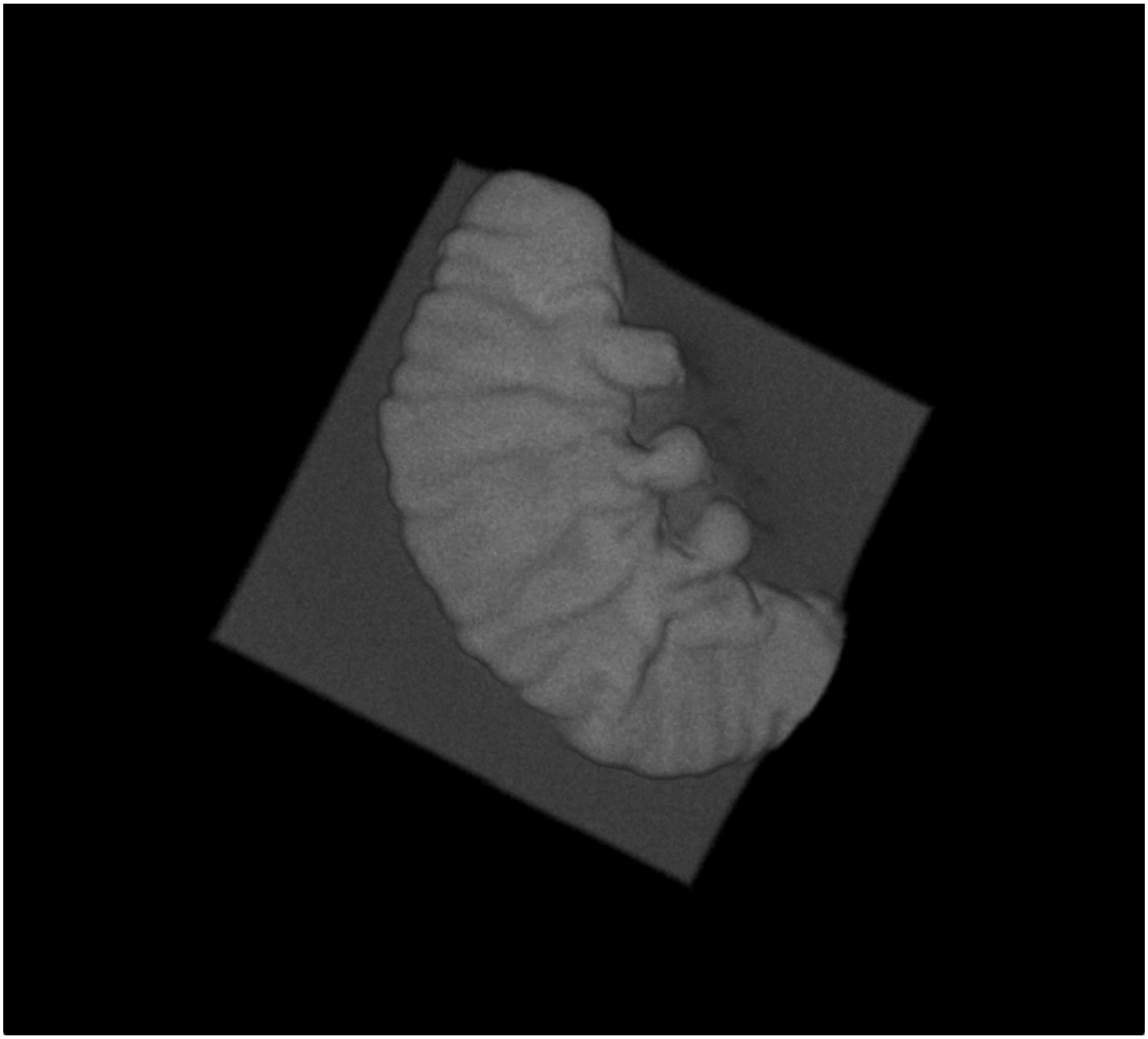

Figure S1. *Shadow Imaging with Argon Laser*. Using the argon laser for excitation during shadow imaging resulted in significant signal depletion near the slide, leading to renderings with significant added volume near the slide contact surface of imaged specimens.

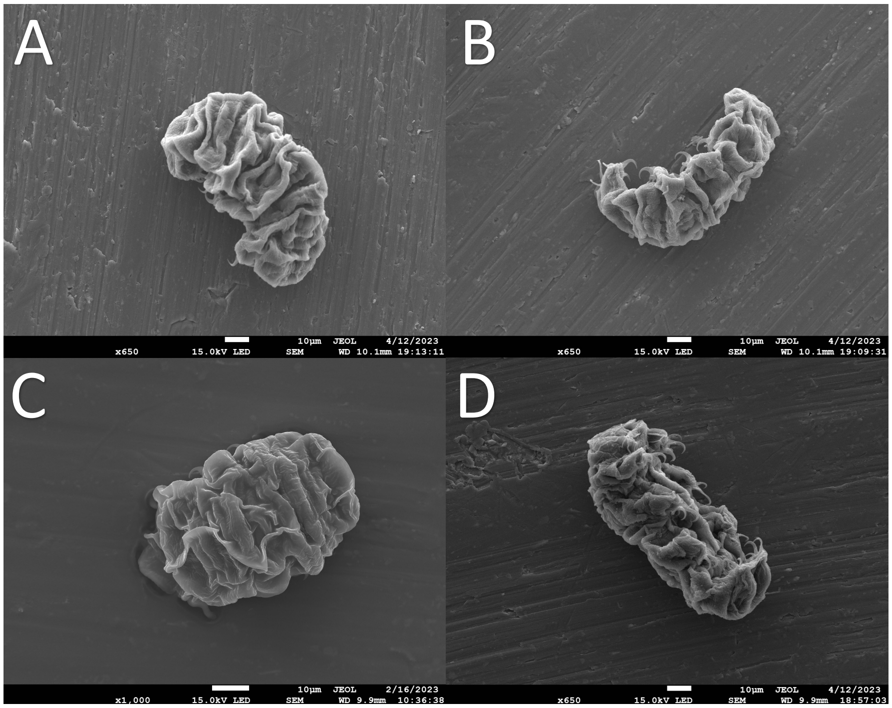

Figure S2. *Structural Deformation of Tardigrades During SEM*. A – Hydrated specimen; B – Second hydrated specimen; C – Sucrose-induced tun; D – $CaCl_2$-induced tun. All hydrated specimens displayed apparent structural collapse while only a few sucrose-induced and $CaCl_2$-induced tuns did.

**State**	**Average Volume (μm^3)**	**Percent Difference to Hydrated**
**Hydrated**	322000	0
**Sucrose-Induced Tun**	111000	65.5
**$CaCl_2$-Induced Tun**	162000	49.7

Table S1. *Percent Changes in Volume for Tun States*. Percent difference was calculated as the difference in average volume between the hydrated and tun samples, divided by the average volume of the hydrated samples. Data was rounded to three significant digits.

**Tardigrade**	**Hydrated Volume (μm^3)**	**Sucrose Tun Volume (μm^3)**	**Percent Difference**
**1**	579000	161000	72.2
**2**	336000	104000	69.0
**3**	568000	227000	60.0
**4**	287000	98000	65.9
**5**	863000	174000	80.0
**Average**	526600	152800	69.4

Table S2. *Single-Tardigrade Measurements*. Given that tardigrades are heterogenous in size, it was tested if single specimens would exhibit the same changes in volumes that were observed in the aggregate experiments (i.e., do single specimens demonstrate the same differences in volume between hydrated and tun states that are observed when different specimens are measured between the two states). The volumes of individual specimens in their sucrose-induced tun states were measured before being rehydrated and then their volumes remeasured. The percent difference between each specimen's hydrated and tun state were calculated and then averaged to allow comparison to the aggregate measurements. Using single specimens, the average volume change from sucrose-induced tuns referenced to their hydrated volumes was 69.4%, nearly matching the change observed in the aggregate measurements of 65.5% (Table S3). This indicates that measuring different specimens for hydrated and tun states is justifiable, preventing the need for attempting the measure the same specimen multiple times (which is challenging given it requires plating one specimen at a time). The percent difference was calculated as the difference between the specimen's volume in the hydrated state and the sucrose-induced tun state, divided by the specimen's volume in the hydrated state. Data was rounded to three significant digits.

Tardigrade	Shadow Imaging Volume (μm^3)	Length (μm)	Diameter (Dorsal to Ventral) (μm)	Diameter (Lateral to Lateral) (μm)	Average Diameter (μm)
**Hydrated 1**	169424	137	49	51	50.6
**Hydrated 2**	224677	146	51	44	47.8
**Hydrated 3**	337085	173	40	55	47.8
**Hydrated 4**	125271	161	38	40	39.2
**Hydrated 5**	358941	194	45	65	55.3
**Hydrated 6**	483979	249	64	46	55.2
**Hydrated 7**	336755	224	36	58	47.2
**Hydrated 8**	451319	231	44	58	51.6
**Hydrated 9**	350116	196	56	46	51.0
**Hydrated 10**	390761	222	58	45	51.8
**Sucrose 1**	90605	91	30	63	46.8
**Sucrose 2**	130888	96	35	65	50.5
**Sucrose 3**	111492	99	34	61	47.7
**Sucrose 4**	151661	112	38	70	54.3
**Sucrose 5**	114476	92	36	64	50.1
**Sucrose 6**	98588	75	35	67	51.5
**Sucrose 7**	93879	74	33	68	50.9
**Sucrose 8**	181087	98	41	75	58.2
**Sucrose 9**	68704	80	29	58	43.7
**Sucrose 10**	74829	76	31	51	41.4
**$CaCl_2$ 1**	126099	109	37	61	49.5
**$CaCl_2$ 2**	105164	112	38	56	47.1
**$CaCl_2$ 3**	188392	147	38	65	52.1
**$CaCl_2$ 4**	131423	144	34	48	41.0
**$CaCl_2$ 5**	188618	133	41	70	55.9
**$CaCl_2$ 6**	99549	124	35	49	42.2
**$CaCl_2$ 7**	312817	161	48	62	55.7
**$CaCl_2$ 8**	117639	131	36	46	41.3
**$CaCl_2$ 9**	245477	132	41	73	57.1
**$CaCl_2$ 10**	148561	104	37	58	48.1
**$CaCl_2$ 11**	164255	137	35	65	50.1
**$CaCl_2$ 12**	119327	103	35	65	50.0

Table S3. *Body Parameter Measurements for All Specimens*. All measurements were conducted in the FIJI port of ImageJ. The length and diameter measurements were used for calculating volume estimations for comparison to the shadow imaging method. Length was measured from the anterior end of the specimen to the posterior end (“head to tail”). Dorsal to ventral refers to measuring the diameter from the dorsal surface of the specimen to the ventral surface (“top to bottom” where the bottom is the side from which the limbs protrude). Lateral to lateral refers to measuring the diameter in the plane orthogonal to the dorsal to ventral measurement. For each specimen, one of the two diameter measurements was calculated as the number of slices needed to encompass the whole specimen multiplied by the step size of 500 nm, depending on the orientation of the specimen (as a line measurement couldn’t be used for both dimensions). Volumes were not adjusted for significant digits intentionally to allow for others to use the raw data, if desired.

Tardigrade	Volume (Shadow imaging)	Volume (Using Dorsal to Ventral Diameter)	Volume (Using Lateral to Lateral Diameter)	Volume (Using Average Diameter)
**Hydrated Average**	322833.2445	376011.6659	414027.7878	386740.089
**Sucrose Average**	111621.4438	172769.0194	149999.8493	88482.45339
**$CaCl_2$ Average**	162277.1093	150111.704	376021.7624	249324.1558
**Hydrated 1**	169424.812	263959.2512	288723.9561	276202.8265
**Hydrated 2**	224677.656	301697.406	227752.5904	263427.7174
**Hydrated 3**	337085.406	217999.9566	423579.7812	312332.7292
**Hydrated 4**	125271.727	188156.3404	203029.652	195522.2832
**Hydrated 5**	358941.25	315638.014	648527.0539	467260.0722
**Hydrated 6**	483979.688	805343.5956	424551.3414	599838.9612
**Hydrated 7**	336755.562	228531.2952	605303.0335	394422.7496
**Hydrated 8**	451319.094	360803.9747	631000.1552	486523.5393
**Hydrated 9**	350116.25	487141.7418	326802.8819	402985.1065
**Hydrated 10**	390761	590845.0836	361007.4319	468884.9047
**Sucrose 1**	90605.617	134279.9191	143812.1111	79086.59951
**Sucrose 2**	130888.867	191355.513	163300.0435	96977.74712
**Sucrose 3**	111492.258	180781.3012	147982.6129	89184.95511
**Sucrose 4**	151661.328	261001.3223	217352.2205	130195.3778
**Sucrose 5**	114476.781	188673.3397	150733.6214	91635.46399
**Sucrose 6**	98588.391	149928.541	135703.9868	78956.29489
**Sucrose 7**	93879.758	130623.5466	136422.2497	75642.38805
**Sucrose 8**	181087.344	266758.9783	218249.6432	131556.9262
**Sucrose 9**	68704.516	109483.1435	105839.7892	60214.15712
**Sucrose 10**	74829.578	114804.5889	80602.21425	51374.62422
**$CaCl_2$ 1**	126099.094	120884.8244	326168.8417	211046.9287
**$CaCl_2$ 2**	105164.336	127104.6913	278796.3804	195597.9643
**$CaCl_2$ 3**	188392.594	171908.5062	502341.7607	315495.2228
**$CaCl_2$ 4**	131423.078	131341.4522	263336.0268	191657.1864
**$CaCl_2$ 5**	188618.656	176061.0449	527289.7496	328182.0325
**$CaCl_2$ 6**	99549.031	119688.6492	239614.5736	174500.3955
**$CaCl_2$ 7**	312817.281	298520.4134	503268.008	394248.4042
**$CaCl_2$ 8**	117639.719	133704.0095	224581.0204	176213.3127
**$CaCl_2$ 9**	245477.891	175307.1803	561533.3687	341086.5468
**$CaCl_2$ 10**	148561.375	115019.0095	282528.8531	189520.4623
**$CaCl_2$ 11**	164255.234	132320.2169	459464.2276	271230.7475
**$CaCl_2$ 12**	119327.023	99480.45011	343338.3379	203110.666

Table S4. *Comparison of Volume Measurement Methods*. Volume estimates were conducted as previously described in the literature, estimating the volume of the specimen either as a perfect cylinder (hydrated and $CaCl_2$-induced tuns, $V = \pi r^2 l$ where V is the volume, r is the radius, and l is the length) or a perfect hemicylinder (sucrose-induced tuns, $V = (1/2)\pi r^2 l$ where V is the volume, r is the radius, and l is the length). Three different ways to estimate the volume using three different measurements of diameter are displayed. In the first, the radius is considered as half of the diameter measured from the dorsal to ventral surfaces (except for sucrose-induced tuns, for which the entire dorsal to ventral length was used as the radius due to the dorsal-ventral length consistently being half of the lateral-lateral length). In the second, the radius is considered as half of the diameter measured in the lateral to lateral direction. In the third, the radius is considered as half of the average of the diameters measured dorsal to ventral and lateral to lateral. Volumes were not adjusted for significant digits intentionally to allow for others to use the raw data, if desired.

	**Volume (Using Dorsal to Ventral Diameter)**	**Volume (Using Lateral to Lateral Diameter)**	**Volume (Using Average Diameter)**
**Hydrated (r)**	0.681	0.661	0.939
**Sucrose (r)**	0.973	0.943	0.967
**$CaCl_2$ (r)**	0.915	0.809	0.936

Table S5. *Correlation Coefficients of Volume Estimation Methods to Shadow Imaging Measurements.* Pearson's correlation coefficient, r, between the volumes measured by shadow imaging and the volumes estimated by three different methods was calculated in Microsoft Excel using the CORREL function. The correlations shows that if one wants an accurate estimation of the volume of hydrated or $CaCl_2$-induced tun specimens for comparative measurements, the diameter should be measured as the average diameter.

Movie S1. *360 Degree Rotation of a Hydrated Tardigrade.* A three-dimensional rendering produced in the 3D viewer plugin of the FIJI port of ImageJ is rotated 360 degrees to allow for visualization of the rendering from all angles. The specimen is a hydrated tardigrade (e.g., it has not been exposed to a stress condition that would induce tun formation, but it was anesthetized to allow for imaging).

Movie S2. *360 Degree Rotation of a Tun.* A three-dimensional rendering produced in the 3D viewer plugin of the FIJI port of ImageJ is rotated 360 degrees to allow for visualization of the rendering from all angles. The specimen is a $CaCl_2$-induced tun.
